# Mucinous Adenocarcinoma of Gallbladder Presenting As Acute Calculous Cholecystitis

**DOI:** 10.7759/cureus.14548

**Published:** 2021-04-18

**Authors:** Rahul Gupta, Shreeya Indulkar, Rahul Varshney, Jyoti Gupta, Houssem Ammar

**Affiliations:** 1 Gastrointestinal Surgery, Synergy Institute of Medical Sciences, Dehradun, IND; 2 Histopathology, American Institute of Pathology and Laboratory Sciences, Hyderabad, IND; 3 Anesthesia and Critical Care, Synergy Institute of Medical Sciences, Dehradun, IND; 4 Radiation Oncology, Himalayan Institute of Medical Sciences, Dehradun, IND; 5 Surgery, Sahloul Hospital, Sousse, TUN

**Keywords:** gallbladder cancer, mucinous adenocarcinoma, laparoscopic cholecystectomy, acute calculous cholecystitis

## Abstract

Mucinous adenocarcinoma (MAC) is a rare type of gallbladder cancer accounting for less than 5% of the reported cases. It is characterized by mucin deposition involving more than 50% of the tumor volume. It is a distinct subtype of gallbladder cancer and associated with poor prognosis. Accurate preoperative diagnosis is difficult. Most of the cases are diagnosed incidentally during the histopathological examination of the resected gallbladder. We report the case of a 75-year-old man who presented with right upper abdominal pain, fever, and vomiting for 15 days. Abdominal ultrasound revealed acute calculous cholecystitis for which he underwent laparoscopic cholecystectomy. Histological examination of the gallbladder found ulcerated gallbladder mucosa lined with dysplastic epithelium. The tumor was mainly composed of dysplastic glands floating in the pools of mucin with invasion of the perivascular connective tissue suggestive of MAC. This case highlights the importance of histological examination of gallbladder after routine cholecystectomy.

## Introduction

Gallbladder cancer is the most frequent cancer of the biliary system. The risk factors for the development of gallbladder cancer include long-standing gallstone disease, porcelain gallbladder, choledochal cyst, and ethnicity [1-3]. It is often detected in the advanced stages and is associated with poor prognosis. Histologically, the most frequent type of gallbladder cancer is adenocarcinoma accounting for 80-90% of all cases [2]. The pathological subtypes of adenocarcinoma are pancreatobiliary, papillary, tubular, mucinous, and signet-cell carcinoma [2]. Mucinous adenocarcinoma (MAC) is one of the rarest types of gallbladder cancer. Most of them are incidentally detected on histological examination of the gallbladder [4-6]. Here, we report a case of elderly male presenting with acute calculous cholecystitis who was diagnosed to have MAC on histopathological examination of the gallbladder.

## Case presentation

**Figure 1:**
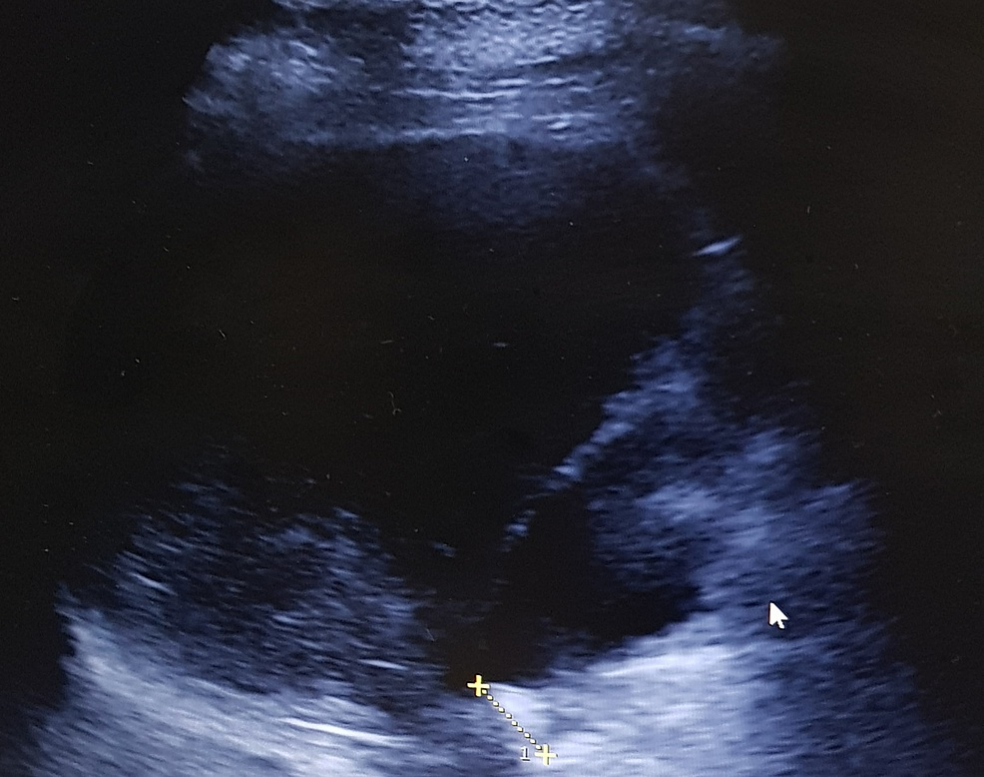
Abdominal ultrasound showing the thickened gallbladder containing echogenic material (arrow) with stone impacted at its neck.

A 75-year-old man presented with a history of right upper quadrant pain, fever, and vomiting for 15 days. Abdominal examination was unremarkable. Gallbladder was not palpable. Blood investigations were within normal limits. Abdominal ultrasound revealed distended, uniformly thickened gallbladder containing echogenic material with a 2.5 cm stone impacted in the neck of the gallbladder (Figure 1). Liver and common bile duct were normal. There was no obvious periportal lymphadenopathy.

On laparoscopic cholecystectomy, there were dense adhesions between the gallbladder and omentum. The gallbladder was overdistended, edematous, and filled with mucous-like material and a 2.5 cm single stone at its neck. The operative time was 120 minutes with blood loss of 100 mL. Postoperative course was uneventful. Tumor markers (carcinoembryonic antigen and carbohydrate antigen 19-9) were within normal limits.

**Figure 2:**
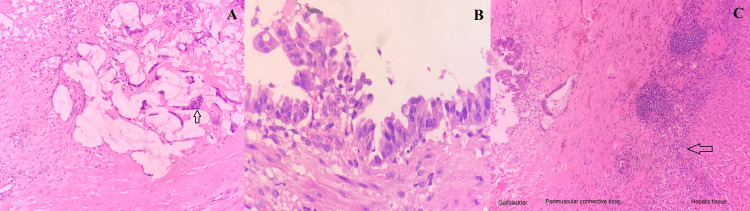
Microscopic examination of the gallbladder showed dysplastic glands (arrow) floating in the mucin pool (HE ×100) (A). The gallbladder mucosa showed dysplastic changes in the epithelium (HE ×400) (B). The tumor was seen infiltrating the peri-muscular connective tissue on the hepatic side (arrow) (HE ×100) (C). HE, hematoxylin and eosin

On gross examination, the gallbladder mucosa was ulcerated with the presence of gray-brown mucus-like solid material. On microscopy, the gray-brown tissue showed predominantly eosinophilic necrotic debris and tumor comprising pools of mucin with a few dysplastic glands floating amidst it. The ulcerated gallbladder mucosa showed dysplastic epithelium with pools of mucin. The tumor was seen invading the perivascular connective tissue on the hepatic side of the gallbladder (Figure 2).

**Figure 3:**
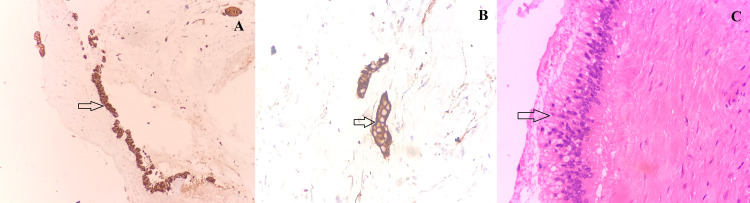
Immunohistochemical analysis showed positive staining of the dysplastic glands of mucinous adenocarcinoma with pancytokeratin (arrows) (A, B). Histological examination of the adjacent gallbladder mucosa revealed gastric-type tall columnar cells (arrow) (HE ×400) (C). HE, hematoxylin and eosin

Immunohistochemical analysis showed positive staining of the dysplastic cells with pancytokeratin (Figure 3). The focally preserved gallbladder epithelium at its neck showed acute-on-chronic cholecystitis (Figure 3). The final diagnosis of MAC of the gallbladder was made with stage pT2bNxMx. The patient denied to undergo completion extended cholecystectomy, and on the last follow-up at two months after surgery, the patient was doing well with no recurrence.

## Discussion

MAC is a rare pathological subtype of adenocarcinoma. It is characterized by stromal mucin deposition involving more than 50% of the tumor [2]. The exact incidence of MAC affecting the gallbladder is not known. In a large series of 606 gallbladder cancers, only 15 (2.5%) cases of MAC were reported [4]. In another series of 987 gallbladder cancers diagnosed by fine needle aspiration, 33 (3.3%) cases were diagnosed to have MAC [1].

The clinical presentation is often similar to that of benign gallstone disease [4]. Both sexes are equally affected with the mean age of 65 years [4]. Most cases are diagnosed on histopathological examination of the resected gallbladder as seen in the present case [4-6]. MAC can be suspected preoperatively with the help of radiological investigations. They appear as irregular, focal gallbladder wall thickening or protruding solid cystic mass from the gallbladder wall on abdominal ultrasound [2,4,7]. Some cases of gallbladder MAC are associated with porcelain gallbladder which is easily diagnosed on computed tomography as mural calcification [7]. On magnetic resonance imaging, the tumor shows moderate enhancement on gadolinium-enhanced T1-weighted gradient-recalled echo images and appears as an irregular papillary lesion arising from the gallbladder wall [8]. However, the tumor is not well visualized on non-enhanced T1 or T2-weighted images. An interesting finding on magnetic resonance cholangiopancreatography is the appearance of linear or curvilinear striations along the long axis of the gallbladder corresponding to the thick mucus bundle labeled as “mucus thread” sign [8]. These striations can also be observed on abdominal ultrasound in some cases [8]. In locally advanced or metastatic cases, ultrasound-guided fine needle aspiration can be performed to diagnose MAC [1].

The treatment of MAC in resectable cases is radical cholecystectomy similar to that of conventional gallbladder cancer. In patients with incidental diagnosis of MAC, completion extended cholecystectomy should be offered for appropriate staging of the disease. In locally advanced cases, palliative chemotherapy or radiation can be offered. The reported overall survival of gallbladder MAC is worse than that of conventional adenocarcinoma [4]. The three-year overall survival reported in the largest series of 15 gallbladder MAC cases was 1% compared to 39% in 567 patients with conventional gallbladder carcinoma [4].

## Conclusions

MAC is a distinct and rare subtype of gallbladder cancer. It is most often detected incidentally and should be included in differential diagnosis while treating elderly patients with acute cholecystitis. The prognosis of MAC is significantly worse compared to that of conventional gallbladder adenocarcinoma.
